# Large scale validation of a new non-invasive and non-contact bilirubinometer in neonates with risk factors

**DOI:** 10.1038/s41598-020-67981-9

**Published:** 2020-07-07

**Authors:** Animesh Halder, Aniruddha Adhikari, Ria Ghosh, Soumendra Singh, Amrita Banerjee, Nilanjana Ghosh, Arnab Madhab Bhattacharya, Shrabani Mandal, Prantar Chakrabarti, Debasis Bhattacharyya, Hatem M. Altass, Moataz Morad, Saleh A. Ahmed, Asim Kumar Mallick, Samir Kumar Pal

**Affiliations:** 1 Technical Research Centre, S. N. Bose National Centre for Basic Sciences, Block JD, Sector-III, Salt Lake, Kolkata, 700106 India; 2 Department of Applied Optics & Photonics, University of Calcutta, Block JD-2, Sector-III, Salt Lake, Kolkata, 700106 India; 3 Department of Chemical, Biological & Macromolecular Sciences, S. N. Bose National Centre for Basic Sciences, Block JD, Sector-III, Salt Lake, Kolkata, 700106 India; 4 Center for Astroparticle Physics and Space Science, Bose Institute, Block EN, Sector-V, Kolkata, 700091 India; 5 Department of Paediatric Medicine, Nil Ratan Sircar Medical College & Hospital, 138, AJC Bose Road, Sealdah, Raja Bazar, Kolkata, 700014 India; 6 Department of Haematology, Nil Ratan Sircar Medical College & Hospital, 138, AJC Bose Road, Sealdah, Raja Bazar, Kolkata, 700014 India; 7 Department of Gynecology & Obstetrics, Nil Ratan Sircar Medical College & Hospital, 138, AJC Bose Road, Sealdah, Raja Bazar, Kolkata, 700014 India; 8 Department of Chemistry, Faculty of Applied Science, Umm Al-Qura University, Makkah, 21955 Saudi Arabia; 9 Department of Chemistry, Faculty of Science, Assiut University, Assiut, 71516 Egypt

**Keywords:** Paediatric research, Risk factors, Biomedical engineering, Paediatrics

## Abstract

The study was aimed to evaluate the performance of a newly developed non-invasive and non-contact bilirubin measurement device (AJO-Neo) as an alternative to the conventional invasive biochemical method of total serum bilirubin (TSB) estimation in preterm and term neonates suffering from hyperbilirubinemia associated with risk factors, and/or undergoing phototherapy. The safety and efficacy of the device were assessed in 1968 neonates with gestational ages ranging from 28 to 41 weeks and suffering from incidences of hyperbilirubinemia. Linear regression analysis showed a good correlation between AJO-Neo and the conventional method of TSB (Pearson’s coefficient, *r* = 0.79). The small bias (0.27 mg/dL) and limits of agreements (− 3.44 to 3.99 mg/dL) were within the range of clinical acceptance. The device was also precise in the measurement of bilirubin levels in all subgroups of the study. The receiver operator curve (ROC), that takes account of both sensitivity and specificity of a device showed high efficacy of the device (area under the curve, AUC = 0.83) in the detection of bilirubin. While monitoring the bilirubin level during phototherapy, the device indicated promising results showing good agreement with TSB. Specificities and sensitivities of the device indicated a much higher accuracy in neonates with associated risk factors for hyperbilirubinemia. Hence, the newly developed device (AJO-Neo) is reliable in measuring bilirubin level in preterm, and term neonates irrespective of gestational or postnatal age, sex, risk factors, feeding behavior or skin color.

## Introduction

**Figure 1:**
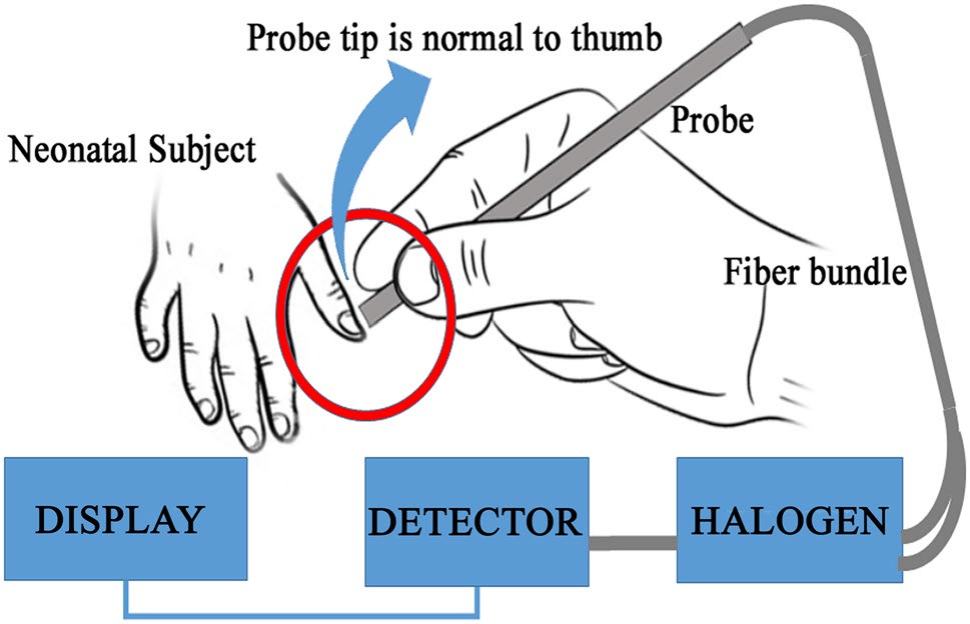
The technique used by the new non-invasive device, AJO-Neo to collect information about bilirubin from the neonatal nail bed. A probe consisting of six illumination fibers and one collection fiber is placed perpendicular to the thumbnail plate as described in our previous study (Ref.^18^). The illumination fiber is connected to the light source i.e., halogen and collection fiber is connected to the detector. Illumination fiber illuminates the sublingual arcade below the nail plate with a particular light dose, collection fiber collects the reflected light (which carries the information from the blood) and carries it to the spectroscopic detector. Indigenously designed program analyses the data, compute the bilirubin level, and displays the value in the monitor.

Neonatal jaundice or *Icterus neonatorum* affects more than 60% of the term and 80% of preterm newborns in the first week of life^1–3^ due to excessive production of bilirubin, and the inability of the newly developed liver to excrete it^4^. Although benign at low concentrations, persistently elevated levels of bilirubin can cause severe neurotoxicity termed as kernicterus, which may lead to significant morbidity and mortality^5,6^. Thus, proper monitoring of the bilirubin level in newborns is mandatory as per the guideline of American Academy of Pediatrics (AAP) to ensure appropriate management^7^. The contemporary method of detection of serum bilirubin concentrations (TSB) involves painful blood sampling^8,9^ which suffers from multiple long term consequences like infection, osteomyelitis (though in rare cases), etc.^9–11^. In this regard, transcutaneous bilirubinometry (TcB) (BiliCheck™^12^, JM-105™^13^, etc. are the commercial versions available) can become an alternative to the repetitive blood sampling^14^ and associated complications^15^. However, certain inherent limitations affect their widespread use in hospital settings^14,16,17^. Particularly, they have been found to be less accurate in Asian, Hispanic and African populations (having black skin tone), during phototherapy and also in babies having hyperbilirubinemia associated with risk factors as designated by AAP (neonates having ABO incompatibility, Rh incompatibility, birth asphyxia, sepsis, G6PD deficiency, etc.)^7^. In our previous study^18^, we observed that the neonatal nail bed could be a potential site for non-invasive estimation of the bilirubin level. A significant correlation was found between the measurements by a newly developed non-invasive bilirubin measurement device (i.e., AJO-Neo) and invasive conventional biochemical method (TSB), even in the subjects who underwent phototherapy, though the sample size was very small. The AJO-Neo device consists of an optical probe having six illumination and one collection fiber connected to a halogen light source and a spectroscopic detector respectively (detailed in Ref.^18^). The illumination fiber illuminates the sublingual arcade below the nail plate with a particular intensity of light, while the collection fiber collects the reflected light (which carries the information from the blood) and carries it to the spectroscopic detector (Fig. 1). An indigenously designed program analyses the data, computes the bilirubin level and displays the value in the monitor. As the device collects data from the blood rather than skin (unlike conventional TcB meters), and analyse the whole absorbance spectrum in order to compute the bilirubin level, it was hypothesized to give accurate measurement values in babies with risk factors and babies undergoing phototherapy.

Thus, the primary aim of this study was to investigate the performance of AJO-Neo in comparison to the conventional biochemical method (TSB) in an Asian population of preterm, near-term and term neonates under routine conditions. Further aim was to assess the effect of risk factors and phototherapy on the accuracy of the device.

## Methods

### Study settings

This was a prospective observational study conducted over 15 months starting from January 2018 at the Department of Pediatric Medicine, Nil Ratan Sircar Medical College and Hospitals (NRSMH, a Govt. aided tertiary hospital), Kolkata, India.

### Sample size estimation

The sample size was estimated using the Everald’s equation for power calculation in diagnostics tests^20^. Assuming the expected lowest sensitivity (SN) to be 95%, lowest expected specificity (SP) to be 80%, confidence interval (W) for both sensitivity and specificity to be 5% and prevalence of neonatal jaundice to be 15%^21^ the minimal sample size required to achieve the targeted sensitivity and specificity were found to be 487 and 290 respectively. However, we decided to include a much higher number (N = 1968) of subjects in our study to reach a more robust statistical outcome. Using a similar method, the minimum sample size required for assessment during phototherapy was found to be 59 (assuming 100% of them are undergoing phototherapy and W to be 5%), to reach the targeted sensitivity of 96%. We evaluated 68 samples in this subcategory.

### Study design and subjects

The study included 2092 neonates^22^ with gestational age from 28 to 40 weeks. Among them, 29 subjects failed the recruitment criteria, of which 20 subjects had cannula on either of the hands and 9 patients had other complications (e.g., inaccessible thumbnail, uneven nail bed, or other physical problems with the thumb), and were thus excluded. Based on deteriorated blood samples (haemolysed blood samples, delayed blood processing, inadequate blood volume, and ambiguous blood information), 95 patients were further ruled out from the analysis. Hence, the effective population size of 1968 neonates was considered for the study. Among them, 261 subjects belonged within the category of AAP described risk factors, namely ABO incompatibility, Rh incompatibility, birth asphyxia, sepsis, and G6PD deficiency while the rest were idiopathic. In this context, anticipating the erratic measurement result we excluded the neonates who were hypotensive. Comprehensive details of the subjects are provided in Table 1. Particulars about inclusion and exclusion criteria are described in Supplementary Table S1. The risk factors are defined in Supplementary Table S2.

**Table 1:** Demographic details of the new-born subjects participated in the study.

**Description**		**Number of Subjects**
Neonates (N)		1968
TcB measurement (n)		1968
Mode of delivery	Spontaneous vaginal	590
	Assistive vaginal	787
	Elective C-section	394
	Emergency C-section	197
Gestational Age (wk)	< 35	435
	35–37^6/7^ (%)	558
	38–39^6/7^ (%)	482
	40 (%)	82
	Unknown	411
Birth Weight	Low birth weight (LBW)	708
	Very low birth weight (VLBW)	258
	Extremely low birth weight (ELBW)	45
Gender	Male	1,094
	Female	873
	Ambiguous (DSD)	01
Race	Asian	1968
Feeding	Breast (%)	1,377
	Formula (%)	197
	Both (%)	394
	Unknown (%)	0
Post-natal age	≤ 24 h	73
	24–47.9 h	206
	48–71.9 h	586
	≥ 72 h	1,103
Phototherapy		1,038
Blood transfusion		18
Disorders	ABO incompatibility	38
	Rh-incompatibility	51
	G6PD deficiency	34
	Sepsis	46
	Birth asphyxia	92
	Others*	1707

*Other disorders include maternal varicella, hypoglycaemia, jitteriness, sclerema, premature rupture of the membranes (PROM), apnoea of prematurity, intrauterine growth-retarded (IUGR), late-onset neonatal sepsis (LONS), gastric duplication cyst, pneumonia on mv, polycythaemia, hepatosplenomegaly, torch (+ Ve, HSV, CMV), congenital rubella, rubella (IgM +), neonatal convulsion, hyperthermia, laboured breathing, hypothyroid, osteogenesis imperfect, meningitis, congenital anomaly, Pierre Robin syndrome, chorioamnionitis, and mother hypothyroid.

The efficacy of the device during phototherapy was evaluated in 68 subjects at 24 h intervals up to 72 h with recommended guidance related to the initiation of phototherapy in preterm infants^23^. In the gestational age based analysis, the effective sample size was 1557 (as the gestational age of 411 subjects were unknown), which is sufficiently higher than the minimum sample size required to conclude the efficacy of the device.

It is worth mentioning that the recruitment of neonates was not consecutive as not all physicians practicing in the department were involved in the study. The neonates getting treatment under the physicians associated with the study were inducted. The appearance of possible selection bias was avoided following the approach described by Hammer et al.^24^. Random assignment of doctors (a general policy for the public hospitals in India), large timeframe of the study (15 months), sufficiently large sample size, collection of data throughout 24 h window, and enough number of subjects in each subcategory (i.e., stratification of samples) helped in avoidance of the sampling bias.

### Quality assurance in data collection

Care was taken that a similar clinical protocol i.e., study, reference, and sample collection methods, and patient enrolment strategies were prospectively maintained throughout the experimental period. To avoid bias in measurements, particular care was taken to keep the technicians, clinicians, investigators, and data analysts at data collection sites blinded to the AJO-Neo and the TSB data. Data of each neonate on pre-defined variables like the date, identification number, sex, gestational age, maternal history, whether having any risk factors, treatment details, etc. was collected from clinical charts on a tablet having required database with the in-built proforma by one laboratory technician hired for the study purpose. Blood collection, serum isolation, and measurements by AJO-Neo were performed by trained nurses of the Department of Pediatric Medicine, NRSMH. They were responsible for uploading the AJO-Neo readings to the database. TSB was measured by expert clinical biochemists at Central Laboratory, NRSMH who were completely unaware of the study. The TSB readings with proper identification numbers of the selected subjects were uploaded by another laboratory technician hired for the study purpose. The readings of both the devices (AJO-Neo and the conventional) were matched based on the identification number by one research staff, to ensure complete blindness of the study. Complete blinding was maintained to keep the two sets of readings separate.

### Bilirubin measurement

For simultaneous measurement, about 2 mL of blood was collected for the conventional TSB test within 30 min of the TcB reading. The TSB of the subjects was quantitatively determined by the 2,5-dichlorophenyldiazonium tetrafluoroborate (DPD) diazo method described by Jendrassik & Groff^25^, using the commercially available test kit (Autospan Liquid Gold, Span Diagnostics, India) within 1 h of blood collection in the Central Laboratory, NRSMH. For the test, serum was first isolated from the collected blood and then examined with the test kit. To prevent the photoreduction of bilirubin the serum samples were carefully kept in the dark at 4 °C before analysis. Guidelines provided by the National Accreditation Board for Testing and Calibration Laboratories (NABL)^26^ were followed to maintain the accuracy and precision of the technique. The coefficient of variation for the hospital laboratory was targeted for < 6%. During the study period, each of the actual variance values, assessed every 3 months, ranged from 3 to 5%.

### Statistical analysis

Analysis of the data was done using descriptive statistical analysis, simple linear regression analysis, and the Bland & Altman method^27–31^. To analyze the accuracy of the device the positive predictive (PPV) and the negative predictive (NPV) values were calculated. The PPV indicates the number of test positives in a population as indicated by the instrument which is also regarded as true positives as indicated by the conventional method. The NPV indicates the proportion of test negatives in a population as directed by the instrument that is also considered as true negatives as indicated by the conventional method. Receiver operative characteristic (ROC) curves were constructed using the method described in previous studies^32–34^ to determine the positive predictive value (PPV), negative predictive value (NPV), sensitivity, and specificity^35^. The cut of values (i.e., the requirement of phototherapy or not) was set considering the 95th, 75th, and 40th percentile of TSB values in the Bhutani nomogram^7,32^. GraphPad Prism 5.0 (GraphPad Software, USA) and SigmaPlot 12.5 (Systat Software, USA) were utilized for the analysis of the data.

### Ethical considerations

For the present work, all necessary ethical permissions were taken from the Institutional Medical Ethics Committee, NRSMH, Kolkata (Ref. No.- No/NMC/26, Dated 03.01.2018). All studies involving human subjects were performed following the Declaration of Helsinki^19^ and guidelines provided by the Indian Council for Medical Research (ICMR), Govt. of India. Written informed consent was obtained from parents or legal guardians who agreed to participate in the study after understanding the details of the study and its consequences. All data and information about the subjects were anonymized kept confidential and used only for this study.

## Results

### Correlation between TSB and AJO-Neo

**Figure 2:**
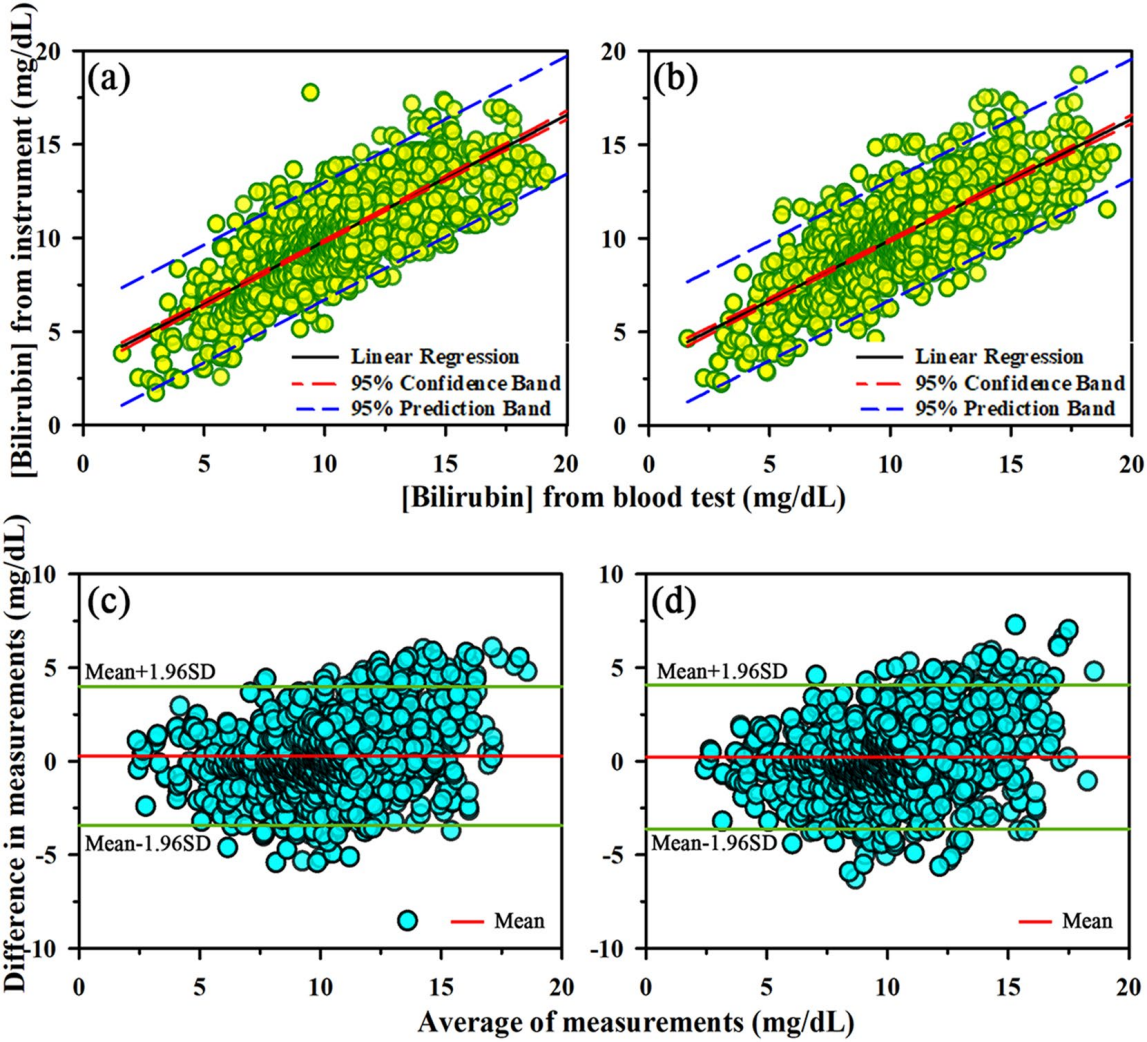
Relationship between paired bilirubin values obtained by AJO-Neo and conventional TSB. Linear regression plots of AJO-Neo versus TSB in the total population (N = 1968) when AJO-Neo data were taken from (**a**) right nail bed and (**b**) left nail bed. Bland–Altman plots (mean and limits of agreement) for the total population (N = 1968) when AJO-Neo data were taken from (**c**) right nail bed and (**d**) left nail bed.

Figure 2 represents the linear regression analysis of the total study population (*N* = 1,968) along with 95% confidence intervals (CI) and 95% prediction intervals. The Pearson’s correlation coefficients (*r*) are calculated as follows: right nail bed, 0.78 (slope 0.64 and intercept 0.23); left nail bed, 0.79 (slope 0.67 and intercept 0.27). These results indicate a strong correlation between the conventional method of TSB estimation and AJO-Neo. Bland–Altman analysis between the two methods (TSB and AJO-Neo) (Fig. 2) reveals a small positive bias for both the nail beds: right, 0.23 mg/dL (95% CI of bias: 0.14 to 0.32 mg/dL) and left, 0.27 mg/dL (95% CI of bias: 0.19 to 0.36 mg/dL) along with SD of 1.89 and 1.96. The 95% limits of agreement (mean ± 2SD) values lie within a range of 3.44 to 4.00 mg/dL (right, − 3.62 to 4.09 mg/dL; left, − 3.44 to 3.99 mg/dL). The 95% limits of agreement depict that 95% of the deviations are expected to lie within the prescribed limit, which in turn determines the efficacy of the instrument.

### Effect of gestational age

The gestational age was not found to be a cofounding factor in regression analysis. The variability in measurement accuracy was not dependent on the gestational age of the neonates (Table 2). Pearson’s correlation coefficient *r* found to be as follows: newborns with gestational age < 35 weeks, right thumbnail, 0.84 (bias, 0.55; limits of agreement, − 3.1 to 3.2) and left thumbnail, 0.81 (bias, 0.01; limits of agreement, − 3.5 to 3.5); newborns with gestational age 35–38 weeks, right, 0.77 (bias, 0.67; limits of agreement, − 3.0 to 4.3) and left, 0.74 (bias, 0.63; limits of agreement, − 3.2 to 4.5); and newborns with gestational age > 38 weeks, right, 0.81 (bias, 0.5; limits of agreement, − 3.2 to 4.2) and left, 0.82 (bias, 0.5; limits of agreement, − 3.5 to 4.3).

**Table 2:** Effect of gestational age in the correlation between bilirubin values obtained from AJO-Neo and TSB and the predictive indices of AJO-Neo.

**Gestational age**	**Statistical Test**	**Parameters**		**Right thumb nail plate**	**Left thumb nail plate**
< 35 weeks	Linear regression analysis	Regression Coefficient (r)		0.84	0.80
		P value		< 0.0001	< 0.0001
		Slope		0.83	0.77
		Intercept		1.52	1.14
	Bland altman analysis	Bias (95% CI)		0.55(− 0.09 to 0.21)	0.002(− 0.17 to 0.17)
		Standard deviation		1.62	1.83
		Limits of agreement		− 3.12 to 3.23	− 3.58 to 3.59
	Accuracy	Above 95th percentile	Sensitivity	0.84	0.69
			Specificity	0.99	0.99
			PPV	0.93	0.89
			NPV	0.98	0.98
		Above 75th percentile	Sensitivity	0.71	0.57
			Specificity	0.97	0.95
			PPV	0.83	0.70
			NPV	0.94	0.91
		Above 40th percentile	Sensitivity	0.88	0.85
			Specificity	0.90	0.88
			PPV	0.80	0.76
			NPV	0.94	0.93
35–38 weeks	Linear regression analysis	Regression Coefficient (r)		0.77	0.74
		P value		< 0.0001	< 0.0001
		Slope		0.64	0.60
		Intercept		3.20	3.69
	Bland altman analysis	Bias (95% CI)		0.67 (0.56 to 0.79)	0.63 (0.51 to 0.75)
		Standard deviation		1.88	1.97
		Limits of agreement		− 3.01 to 4.36	− 3.23 to 4.49
	Accuracy	Above 95th percentile	Sensitivity	0.52	0.58
			Specificity	0.98	0.98
			PPV	0.83	0.80
			NPV	0.94	0.94
		Above 75th percentile	Sensitivity	0.58	0.59
			Specificity	0.94	0.94
			PPV	0.75	0.75
			NPV	0.88	0.88
		Above 40th percentile	Sensitivity	0.72	0.71
			Specificity	0.90	0.90
			PPV	0.86	0.86
			NPV	0.79	0.78
> 38 weeks	Linear Regression Analysis	Regression Coefficient (r)		0.81	0.82
		P value		< 0.0001	< 0.0001
		Slope		0.54	0.52
		Intercept		4.24	4.43
	Bland altman analysis	Bias (95% CI)		0.50 (− 0.11 to 1.12)	0.50 (− 0.11 to 1.11)
		Standard deviation		1.99	1.93
		Limits of agreement		− 3.28 to 4.29	− 3.59 to 4.30
	Accuracy	Above 95th percentile	Sensitivity	0.83	0.73
			Specificity	0.98	0.97
			PPV	0.88	0.90
			NPV	0.90	0.93
		Above 75th percentile	Sensitivity	0.72	0.81
			Specificity	0.96	0.96
			PPV	0.88	0.90
			NPV	0.90	0.83
		Above 40th percentile	Sensitivity	0.79	0.79
			Specificity	0.98	0.98
			PPV	0.98	0.98
			NPV	0.72	0.78

### Comparison of TSB and AJO-Neo in risk factor neonates

**Figure 3:**
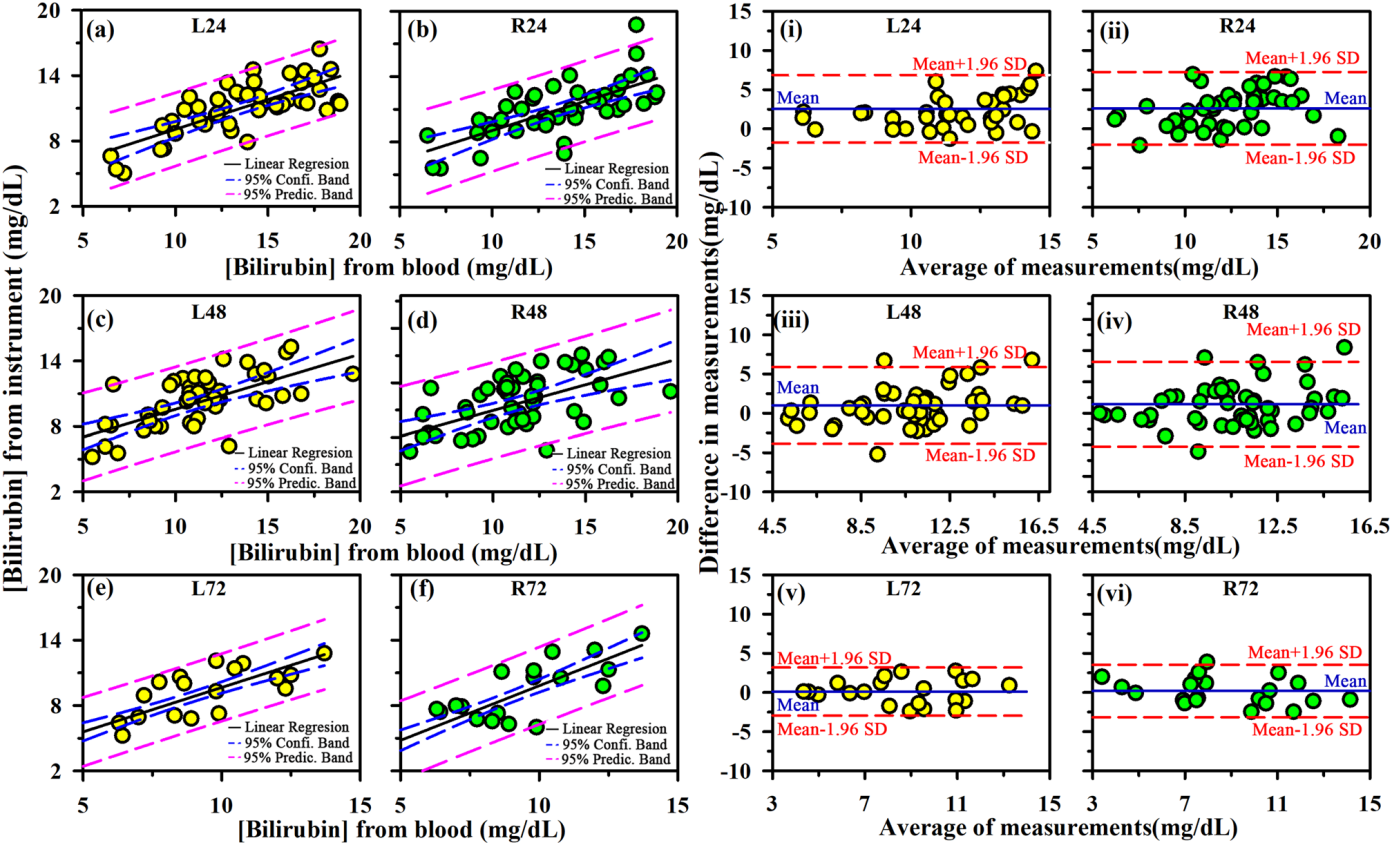
Relationships in matched AJO-Neo and TSB values at different time points of ongoing phototherapy (N = 68). Phototherapy was given following recommendation of AAP based on TSB level, gestational and/or postnatal age, and associated risk factors. Measurements were performed at 24 h of interval. (**a**–**f**) Linear regression plot. (i-vi) Bland–Altman plot. L, left nail bed; R, right nail bed; the numbers (i.e., 24, 48, 72) represents the time of phototherapy.

The performance of AJO-Neo in comparison to the conventional TSB method in severe cases of hyperbilirubinemia associated with risk factors was assessed using linear regression and Bland–Altman analysis. The value of the Pearson’s correlation coefficient (*r*) for neonates suffering from jaundice associated with some risk factors are as follows: birth asphyxia, 0.88 (right nail bed, R) and 0.80 (left nail bed, L); sepsis, 0.87 (R) and 0.78 (L); Rh incompatibility, 073 (R) and 0.71 (L); ABO incompatibility, 0.78 (R) and 0.75 (L); G6PD deficiency, 0.84 (R) and 0.70 (L). Bland–Altman plots for different risk factor babies are shown in Fig. 3. The standard deviation of the AJO-Neo with the conventional TSB varied mostly between 1.57 to 2.03 mg/dL (except Rh incompatibility: right, 2.29; left, 2.37), which is clinically acceptable. Detailed results of comprehensive statistical analysis are presented in Table 3.

**Table 3:** The correlation between bilirubin values obtained from AJO-Neo and TSB along with predictive indices of AJO-Neo in neonates with AAP designated risk factors of hyperbilirubinemia.

**Risk factors**	**Statistical test**	**Parameters**		**Right thumb nail plate**	**Left thumb nail plate**
ABO incompatibility	Linear regression analysis	Regression coefficient (r)		0.78	0.75
		P value		< 0.0001	< 0.0001
		Slope		0.98	0.98
		Intercept		0.84	0.85
	Bland altman analysis	Bias (95% CI)		0.71 (0.47 to 0.96)	0.66 (0.39 to 0.91)
		Standard deviation		1.92	1.98
		Limits of agreement		− 3.04 to 4.48	− 3.31 to 4.63
	Accuracy	Above 95th percentile	Sensitivity	0.85	0.88
			Specificity	0.98	0.98
			PPV	0.85	0.88
			NPV	0.91	0.98
		Above 75th percentile	Sensitivity	0.70	0.85
			Specificity	0.94	0.94
			PPV	0.85	0.85
			NPV	0.87	0.87
		Above 40th percentile	Sensitivity	0.80	0.80
			Specificity	0.91	0.88
			PPV	0.90	0.87
			NPV	0.82	0.81
Rh incompatibility	Linear regression analysis	Regression Coefficient (r)		0.73	0.71
		P value		0.55	0.08
		Slope		0.59	0.55
		Intercept		3.80	4.38
	Bland altman analysis	Bias (95% CI)		0.54 (− 0.10 to 1.18)	0.411 (− 0.25 to 1.07)
		Standard deviation		2.29	2.37
		Limits of agreement		− 3.96 to 2.84	− 3.24 to 2.06
	Accuracy	Above 95th percentile	Sensitivity	0.00	0.16
			Specificity	1.00	1.00
			PPV	0.00	1.00
			NPV	0.88	0.90
		Above 75th percentile	Sensitivity	0.60	0.60
			Specificity	0.92	0.95
			PPV	0.66	0.75
			NPV	0.90	0.90
		Above 40th percentile	Sensitivity	0.80	0.65
			Specificity	0.93	0.87
			PPV	0.88	0.76
			NPV	0.87	0.79
G6PD deficiency	Linear regression analysis	Regression Coefficient (r)		0.84	0.70
		P value		0.55	0.08
		Slope		0.47	0.50
		Intercept		5.48	5.15
	Bland altman analysis	Bias (95% CI)		0.03 (− 0.61 to 0.69)	0.16 (− 0.73 to 0.40)
		Standard deviation		1.87	1.57
		Limits of agreement		− 3.63 to 3.71	− 3.26 to 2.92
	Accuracy	Above 95th percentile	Sensitivity	0.75	0.75
			Specificity	0.90	0.93
			PPV	0.50	0.60
			NPV	0.96	0.96
		Above 75th percentile	Sensitivity	0.70	0.60
			Specificity	0.91	0.91
			PPV	0.77	0.75
			NPV	0.88	0.84
		Above 40th percentile	Sensitivity	0.73	0.86
			Specificity	0.73	0.84
			PPV	0.68	0.81
			NPV	0.77	0.88
Sepsis	Linear regression analysis	Regression Coefficient (r)		0.87	0.78
		P value		0.02	0.19
		Slope		0.82	0.65
		Intercept		0.93	2.85
	Bland altman analysis	Bias (95% CI)		0.88 (0.43 to 1.34)	0.80 (0.22 to 1.39)
		Standard deviation		1.53	1.96
		Limits of agreement		− 2.11 to 3.88	− 3.04 to 4.65
	Accuracy	Above 95th percentile	Sensitivity	0.5	0.16
			Specificity	1.0	1.0
			PPV	1.0	1.0
			NPV	0.93	0.88
		Above 75th percentile	Sensitivity	0.71	0.71
			Specificity	1.00	1.00
			PPV	1.00	1.00
			NPV	0.95	0.95
		Above 40th percentile	Sensitivity	0.63	0.63
			Specificity	0.91	0.94
			PPV	0.70	0.70
			NPV	0.88	0.89
Birth asphyxia	Linear regression analysis	Regression Coefficient (r)		0.88	0.80
		P value		0.42	0.40
		Slope		0.69	0.58
		Intercept		2.78	3.94
	Bland altman analysis	Bias (95% CI)		0.37 (0.01 to 0.74)	0.39 (− 0.01 to 0.79)
		Standard deviation		1.76	1.93
		Limits of agreement		− 3.07 to − 2.44	− 3.40 to 4.18
	Accuracy	Above 95th percentile	Sensitivity	0.73	0.73
			Specificity	1.00	0.98
			PPV	1.00	0.91
			NPV	0.95	0.95
		Above 75th percentile	Sensitivity	0.87	0.77
			Specificity	0.91	0.96
			PPV	0.84	0.92
			NPV	0.93	0.89
		Above 40th percentile	Sensitivity	0.88	0.83
			Specificity	0.91	0.91
			PPV	0.90	0.90
			NPV	0.90	0.86

### Performance during phototherapy

**Figure 4:**
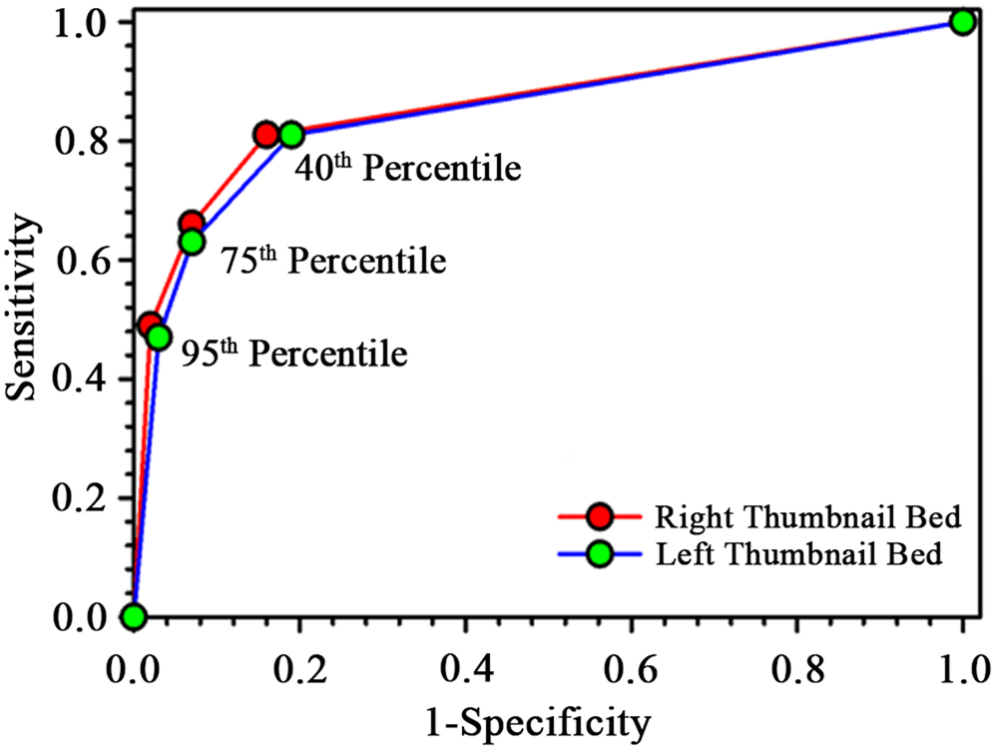
Receiver operating characteristics (ROC) curves for AJO-Neo bilirubin value collected from the left and right nailbed. The cut-off values were 95th, 75th, and 40th percentile of Bhutani nomogram for phototherapy in neonatal hyperbilirubinemia.

To evaluate the performance of AJO-Neo during phototherapy, 68 neonates were monitored for 72 h at regular intervals of 24 h. However, the total number of neonates who underwent phototherapy and subjected to evaluation (not monitored at regular intervals) by AJO-Neo was 1,038. AJO-Neo showed a significantly high correlation coefficient at all the intervals (N = 68) as showed in Fig. 3. Pearson’s correlation coefficients (*r*) are found to be as follows: 24 h, 0.74 (R) and 0.70 (L); 48 h, 0.65 (R) and 0.58 (L); 72 h, 0.82 (R) and 0.83 (L). The Bland–Altman plot (Fig. 4) reveals SD as follows: 24 h, 2.20 (R) and 2.36 (L); 48 h, 2.48 (R), and 2.75 (L); 72 h, 1.56 (R) and 1.74 (L). A complete statistical analysis is presented in Table 4.

**Table 4:** The correlation between bilirubin values obtained from AJO-Neo and TSB at different times of phototherapy (N = 68).

**Hours of phototherapy**	**Statistical test**	**Parameters**	**Right thumb nail plate**	**Left thumb nail plate**
24 h	Linear regression analysis	Regression Coefficient (r)	0.74	0.70
		P value	0.81	0.55
		Slope	0.54	0.53
		Intercept	3.59	3.67
	Bland Altman Analysis	Bias (95% CI)	2.56 (1.90 to 3.22)	2.62 ( 1.91 to 3.33)
		Standard deviation	2.20	2.36
		Limits of agreement	− 1.75 to 6.88	− 2.00 to 7.25
48 h	Linear regression analysis	Regression Coefficient (r)	0.65	0.58
		P value	0.32	0.05
		Slope	0.50	0.47
		Intercept	4.50	4.75
	Bland altman analysis	Bias (95% CI)	1.01 (0.30 to 1.71)	1.15 (0.37 to 1.93)
		Standard deviation	2.48	2.75
		Limits of agreement	− 3.86 to 5.88	− 4.24 to 6.55
72 h	Linear regression analysis	Regression Coefficient (r)	0.82	0.83
		P value	0.05	0.05
		Slope	0.81	1.00
		Intercept	1.49	− 0.20
	Bland altman analysis	Bias (95% CI)	0.11 (− 0.37 to 0.60)	0.18 (− 0.35 to 0.71)
		Standard deviation	1.56	1.74
		Limits of agreement	2.96 to 3.18	− 3.17 to 3.53

### Receiver operator curve (ROC)

The area under the curve (AUC) for the ROC considering all subjects found to be 0.83. (Fig. 4) Considering the 40th percentile of Bhutani nomogram as the threshold value, the specificity and sensitivity found to be 84% and 81% (Table 5). Both PPV and NPV were above 80%. While at 75th percentile sensitivity decreased to 66% with an increase in specificity (93%). The sight of data collection (i.e., right or left nail bed) had an insignificant effect on the AUC. The AUC for different risk factors were as follows: ABO incompatibility, 0.88; Rh incompatibility, 0.85; sepsis, 0.80; G6PD deficiency, 0.79; birth asphyxia, 0.92 (Table 2). A comprehensive result of the ROC curve analysis of different subgroups of the study is provided in Table 2.

**Table 5:** The correlation between bilirubin values obtained from AJO-Neo and TSB along with predictive indices of AJO-Neo for the entire study population (N = 1968).

**Statistical Tests**	**Parameters**		**Right thumb nail plate**	**Left thumb nail plate**
Linear regression analysis	Regression Coefficient (r)		0.78	0.79
	P value		< 0.0001	0.0002
	Slope		0.64	0.67
	Intercept		0.23	0.27
Bland altman analysis	Bias (95% CI)		0.23 (0.14 to 0.31)	0.27 (0.19 to 0.36)
	Standard deviation		1.89	1.96
	Limits of agreement		− 3.62 to 4.08	− 3.44 to 3.99
Accuracy	Above 95th percentile	Sensitivity	0.49	0.47
		Specificity	0.96	0.97
		PPV	0.81	0.91
		NPV	0.91	0.75
	Above 75th percentile	Sensitivity	0.66	0.63
		Specificity	0.93	0.93
		PPV	0.78	0.79
		NPV	0.85	0.87
	Above 40th percentile	Sensitivity	0.81	0.81
		Specificity	0.84	0.81
		PPV	0.85	0.79
		NPV	0.80	0.83

## Discussion and conclusions

The conventional TcB meters are yet to replace the invasive method of blood sampling because the TcB measured by them consists of the extravascular bilirubin (~ 99% contribution) which is completely a different physiological parameter in comparison to TSB. The unpredictable process that regulates the dynamics of bilirubin in the extravascular space makes a 1 to 1 comparison of TSB and TcB impossible^16,17^. Confinement of the measurement volume only to the intravascular space could help in overcoming the problem^17^. The AJO-Neo device is based on such a spectroscopy-based approach where the majority of the information is collected from the vascular bed underneath the nail plate^18^. Thus, we predicted that AJO-Neo will be able to overcome the limitations of the conventional TcB meters i.e., their limited accuracy in preterm infants^34,36–38^, dark skin babies^14,39–41^, neonates having phototherapy^39,42–47^, newborns with low birth weight^48,49^, exclusively breastfed infants^34,50–52^, neonates with higher postnatal age^34^ and high TSB value^34,53–55^, etc.

Our results suggest that the bilirubin value obtained from AJO-Neo has a strong positive linear correlation (Pearson’s *r* = 0.79) with TSB. Multivariate regression analysis showed gestational age, dark skin color (or variation within Indian subpopulation), postnatal age, exclusive breastfeeding, ABO and Rh incompatibility, birth asphyxia, G6PD deficiency, and sepsis are not associated with any variation in the performance of the device. There was also no significant difference in measurement performed on the right or left nail bed. AJO-Neo showed significant agreement with the conventional TSB when measurements were performed during phototherapy. However, the device slightly underestimated bilirubin value when TSB exceeded 15 mg/dL. Otherwise, it marginally overestimated the bilirubin value in all substrata of analysis. Although this may result in unnecessarily prolonged hospitalization and invasive blood sampling, it eliminates the chances of serious clinical errors like not treating a diseased infant and hence decreases infant morbidity and mortality. The AUC of 0.83 (which considers both sensitivity and specificity) in ROC analysis is excellent not only for a non-invasive device but even for an invasive device.

The correlation coefficient between AJO-Neo and TSB measurements of 0.79 that we found in our study is better than those found in Ref.^56–65^ but lesser than Ref.^1,39,61,66–68^. It has to be noted that, the studies where TcB meters showed better correlation were predominantly performed on the white population in which conventional TcB meters generally show good efficacy. Furthermore, the bias and limits of agreements for AJO-Neo were comparable to or better than these studies. Several studies reported that TcB meters significantly overestimate bilirubin levels in dark skin populations like Hispanic, Asian, and African^14,39,40^. For example, Maisels et al.^39^ showed that in black infants, TcB was overestimated by ≥ 3 mg/dL in 17.4% and by ≥ 4 mg/dL in 6.7% subject. Olusanya et al.^34^, reported TcB overestimation by ≥ 2 mg/dL in 64.5%, ≥ 3 mg/dL in 42.7% and ≥ 4 mg/dL in 25.7% of the subjects . In our study values of overestimation by ≥ 2 mg/dL, ≥ 3 mg/dL and ≥ 4 mg/dL were found to be 25%, 4.2% and 0.9% respectively. The considerably less bias and limits of agreements (with better correlation coefficient) further suggest that different tribes or shades of dark skin color have little or no effect on AJO-Neo performance. To the best of our knowledge, no TcB device has been developed to address bilirubin overestimation in the black population, and a low-cost, non-invasive, point of care device for these ethnic groups holds promise for low- and middle-income countries^34^.

In some of the studies, even with a caucasian population, gestational and/or postnatal age has found to be a confounding factor in TcB measurements because of the differences in the maturity and thickness of the skin at different time points^36,37,69,70^. Particularly in preterm babies, the performance of the TcB meters deteriorates^36,38^. Conversely, in our study, both the gestational and postnatal age have no impact on the accuracy of the measurements. It is worth mentioning that, unlike other TcB meters, AJO-Neo showed better correlation (*r* = 0.84) in preterm neonates.

Exclusive breastfeeding or feeding behavior has been found to have no effect on the bilirubin estimation by AJO-Neo though an association between exclusive breastfeeding and TcB overestimation (even overestimation of > 4 mg/dL) is reported earlier^50–52^. Itoh and colleagues^51^, for example, proposed that increased unconjugated bilirubin in breastfed infants may be underpinned by the high enterohepatic circulation of unconjugated bilirubin from deconjugation by β-glucuronidase of the conjugated bilirubin in meconium, which in turn result into the observed higher TcB/TSB discrepancy. As AJO-Neo collects the majority of the information from intravascular space^18^, the aforementioned changes in breastfed babies have a negligible impact on the measurement.

Almost all previous studies have shown the inaccuracy of commercially available bilirubinometers in the clinical setting of phototherapy^39,42–46^. For example, Grabenhenrich et al.^42^, have found that the commercially available TcB meters significantly underestimates bilirubin level (mean bias, − 0.6 mg/dL; limits of agreement, − 4.4 to 3.6 mg/dL) during phototherapy. Hulzebos and colleagues^45^ have reported further underestimation (mean bias, − 3.5 mg/dL; limits of agreement, − 6.8 to − 0.2 mg/dL). The underestimation of the bilirubin level in neonates under phototherapy can lead to severe error in clinical decision making, and eventually, result in critical illness due to undertreatment. At a biochemical level, this phenomenon of bilirubin underestimation can readily be explained by the decrease of skin bilirubin levels by phototherapy (skin bleaching)^45,71–73^. Interestingly, in this study, we found that unlike other non-invasive instruments, AJO device was accurate enough (for example at 24 h of phototherapy: Pearson’s *r*, 0.74; bias, 2.56; limits of agreement, − 1.75 to 6.88 and at 72 h: Pearson’s *r*, 0.82; bias, 0.11; limits of agreement, − 0.37 to 0.60) to predict the bilirubin value during phototherapy. However, we cannot adequately explain the reason behind the low correlation (*r*, 0.65) in the subjects at 48 h and higher correlation (*r*, 0.82) at 72 h of phototherapy but speculate that dynamic processes occurring during the phototherapy, such as changes in hemoglobin or other analyte concentration, may partially account for this phenomenon. However, a more detailed study is required to have a better understanding. Nonetheless, this is a significant technological advancement that can help clinicians to better monitor the phototherapy treatment without performing repetitive painful invasive blood sampling in newborns.

This is one of the few studies that extensively assessed the performance of a non-invasive bilirubinometer in neonates with different risk factors associated with severe hyperbilirubinemia along with gestational and postnatal age. AUC is a parameter which cumulatively describes the sensitivity and specificity of an instrument. Even, for an invasive device AUC more than > 0.80 is considered excellent^74^. Thus, the observed overall AUC of 0.83 is very good for a non-invasive device measuring dynamic factor-like bilirubin. In the case of preterm babies, the AUC was 0.92, followed by 0.88 in term and 0.82 in near term infants further describes the ability of AJO-Neo to have an accurate measurement both in terms of sensitivity and specificity irrespective of gestational age. AUC > 0.80 in all risk factor babies also depicts its usefulness in them.

We think that the strength of this study was the prospective design, a large number of sample in each substratum of analysis, the intraracial variation in dark skin color, the parallel measurement by two methods (estimation of bilirubin by routine biochemical test and AJO-Neo), and collection of TSB by regular nurses for clinical use rather than specifically for a study in which conditions might be optimized. The conventional measurements were performed by the experienced clinical biochemists and laboratory technicians of a tertiary care hospital, reducing the possibility of operator error. Overall, we believe that our results provide a robust estimation of the accuracy of non-invasive bilirubin measurement by a new device and the sources of error that are applicable to routine clinical settings.

Our study has some limitations which further unlocks the possibilities of future trials. Firstly, the study was performed on subjects having a TSB report not more than 20 mg/dL as the subjects found to have severe consequences including exchange blood transfusion. In this regard, it is worth mentioning that, the commercially available transcutaneous bilirubinometers also measure TcB value up to 20 mg/dL^53,75^. Therefore, one recommendation based on the current study should be to use the device with caution when obtaining readings approaching this level. Studying the population having higher TSB and manipulation of the intensity of illumination can further increase the accuracy of the device. Secondly, the results of the noninvasive measurements (AJO-Neo) were not used for clinical management. A detailed study using these readings for real-time clinical management may enlighten its ability to reduce painful blood sampling in day to day clinical practice. Though the sample size was adequately large, another limitation of this study is that it was performed on the Indian population of one center. A detailed multicentric study involving a multiracial population could increase the acceptability of the instrument.

The new non-invasive and non-contact device measured TcB levels accurately irrespective of gestational or postnatal age, sex, birth weight, and feeding behavior in neonates with or without risk factors, even during phototherapy. Only TSB > 15 mg/dL was found to be a confounding factor, which could be eliminated by slight modification in the illumination intensity of the probe. Successful evaluation in babies with risk factors associated with severe neonatal hyperbilirubinemia could open a new era in non-invasive bilirubin detection. The non-invasive and non-contact device offers significantly high sensitivity and specificity in the case of the neonates with risk factors. Interestingly, in this study, we also found the device gave accurate results to predict the bilirubin levels during phototherapy that can help clinicians to better monitor the decrease in bilirubin concentration, and reduce the frequency of painful blood sampling. Moreover, the device uses nail bed as the area of interest which contains less melanin irrespective of the race that in turn helped it to overcome the problem of the aforementioned hazardous pigment. Overall, our results suggest that screening with the current version of AJO-Neo might be most effective at conditions when most TSB levels are expected to be, < 20 mg/dL, and the device can overcome the other limitations of the conventional transcutaneous bilirubinometers.

## Supplementary information

Supplementary information
