# Supplementary material for: Large scale validation of a new non-invasive and non-contact bilirubinometer in neonates with risk factors
Source: Sci Rep. 2020 Jul 7;10:11149. doi: 10.1038/s41598-020-67981-9 (PMC7341797; doi:10.1038/s41598-020-67981-9)
Supplement: Supplementary file 1 — Supplementary information [file 41598_2020_67981_MOESM1_ESM.doc]

**Large scale Validation of a New Non-Invasive and Non-Contact Bilirubinometer in Neonates with Risk Factors**

**Supplementary Information**

Animesh Halder^1,2,‡^, Aniruddha Adhikari^3,‡^, Ria Ghosh^1^, Soumendra Singh^1,4^, Amrita Banerjee^1^, Nilanjana Ghosh^5^, Arnab Madhab Bhattacharya^5^, Shrabani Mandal^5^, Prantar Chakrabarti^6^, Debasis Bhattacharyya^7^, Hatem M. Altass^8^, Moataz Morad^8^, Saleh A. Ahmed^8^, Asim Kumar Mallick^5,*^ and Samir Kumar Pal^1,3,*^

^1^Technical Research Centre,

S. N. Bose National Centre for Basic Sciences, Block JD, Sector-III, Salt Lake, Kolkata: 700106, India

^2^Department of Applied Optics & Photonics,

University of Calcutta, Block JD-2, Sector-III, Salt Lake, Kolkata: 700106, India

^3^Department of Chemical, Biological & Macromolecular Sciences,

S. N. Bose National Centre for Basic Sciences, Block JD, Sector-III, Salt Lake, Kolkata: 700106, India

^4^Center for Astroparticle Physics and Space Science,

Bose Institute, Block EN, Sector-V, Kolkata: 700091, India

^5^Department of Paediatric Medicine,

Nil Ratan Sircar Medical College & Hospital, 138, AJC Bose Road, Sealdah, Raja Bazar, Kolkata: 700014, India

^6^Department of Haematology,

Nil Ratan Sircar Medical College & Hospital, 138, AJC Bose Road, Sealdah, Raja Bazar, Kolkata: 700014, India

^7^Department of Gynecology & Obstetrics,

Nil Ratan Sircar Medical College & Hospital, 138, AJC Bose Road, Sealdah, Raja Bazar, Kolkata: 700014, India

^8^Department of Chemistry, Faculty of Applied Science,

Umm Al-Qura University, 21955, Makkah, Saudi Arabia

**‡ both the authors contributed equally.**

*Corresponding authors: Prof. (Dr.) Samir Kumar Pal, *Senior Professor*

S. N. Bose National Centre for Basic Sciences, Block JD, Sector-III, Salt Lake, Kolkata: 700106, India

Contact: +91-33-3355706/7/8 Ext 230

Email: [skpal@bose.res.in](mailto:skpal@bose.res.in)

Prof. Asim Kumar Mallick, *Professor*

Nil Ratan Sircar Medical College & Hospital, 138, AJC Bose Road, Sealdah, Raja Bazar, Kolkata: 700014, India

Contact: +91-9830008057

Email: [drasimmallick@gmail.com](mailto:drasimmallick@gmail.com)

**Supplementary Table S1:** Inclusion and exclusion criteria for the study.

| **Inclusion criteria** | Inclusion criteria comprised of all the neonates irrespective of the gestational age admitted to the Department of Paediatric Medicine, NRSMH with or without jaundice and those requiring phototherapy to monitor the reduction of bilirubin concentration in the blood with the response to the phototherapy and whose parents were willing to provide written informed consent after getting detailed information about the study. |
| --- | --- |
| **Exclusion criteria** | 1. TSB is higher than 20 mg/dL. 2. Neonates having cannula in either of the hands.   (Due to the presence of cannula or channel the blood circulation in the nailbed may be affected, which might have an impact in the reading by AJO-Neo. As we were comparing the differences between to nailbeds (right and left) we made it exclusion criteria).   1. Neonates who are extremely sick and from whom blood samples cannot be drawn. 2. Babies having major congenital malformation. 3. Neonates having lower peripheral circulation, hypotensive |

**Supplementary Table S2:** Definition of different risk factors associated with neonatal hyperbilirubinemia.

| **Risk Factors** | **Definition** |
| --- | --- |
| **Birth asphyxia** | If there was a presence of at least one of the following condition:   1. Gasping or ineffective breathing or lack of breathing at one minute of life 2. Need for positive pressure Ventilation for >1 minute 3. Apgar score <3 at 5 minutes or longer   [*according to the definition mentioned in Facility Based Newborn Care by Government of India*] |
| **ABO incompatibility** | If there was a mismatch between the blood groups of the mother and the newborn. |
| **Rh incompatibility** | If there was a mismatch between the Rh factors of the mother and the newborn. |
| **G6PD deficiency** | If G6PD levels in blood were <4.6 U/gm hemoglobin. |
| **Sepsis** | Neonatal sepsis has been defined as a clinical syndrome characterized by signs and symptoms of infection with or without accompanying bacteremia in the first month of life. |
